# The Death of the Monograph?

**DOI:** 10.1007/s12109-022-09885-2

**Published:** 2022-05-05

**Authors:** Philip Shaw, Angus Phillips, Maria Bajo Gutiérrez

**Affiliations:** Oxford International Centre for Publishing, Oxford Brookes University, Headington Hill Hall, Oxford, OX3 0BP UK

**Keywords:** Monograph, University press, Open access, Digital, Ebook

## Abstract

A survey of English language academic publishers in the UK, Europe and North America was undertaken in 2021. The objective was to gather data on the current landscape of academic monograph publishing in the arts, humanities, and social sciences and to identify trends. Respondents were asked about their monograph publishing activities, sales, distribution, and about the future direction of their programmes. The paper offers independent analysis of publisher information that may be helpful in informing the debate among stakeholders as to the future of the publication of long-form research in the arts, humanities and social sciences. The results offer key insights into the growth in output of titles, the level of print sales, the move towards open access, usage of monographs, and their pricing.

The demise of the academic monograph has long been predicted, yet long-form research is still with us. The monograph maintains its position as a marker of research quality in the publication record of researchers in the arts, humanities and social sciences (AHSS), still important for decisions around tenure and promotion. It is regarded as an example of excellence in the publication of high-quality research, and demand from researchers to be published in book form remains high.

This paper aims to examine the health of the monograph through the lens of the academic publishing house. The many threats to the monograph include the switch to journal publication by much of the academy, the decline in library spending on print, declining sales per title, and high pricing by publishers both in print and ebook form. A notable recent trend is the arrival of the short-form monograph, which has succeeded in both digital and print formats. Digital and on demand printing are widely in use, enabling shorter runs and reprinting to order for print editions. Legitimate concerns over access, given the high prices of individual volumes, are met to some extent by the presence of monographs in aggregated libraries of content available in digital form.

The present research was designed to examine developments in the area of AHSS monograph publishing, using data collected from publishers. Whilst not providing a perspective from other stakeholders such as researchers, funding bodies and librarians, it does give a valuable insight into the present health of monograph publishing. As the requirements around open access are increasingly to be applied to book as well as journal publication, this is a good time to be collecting relevant data. The results presented also cover the first year of the global pandemic, when there was increased demand for digital resources.

## The Background

The monograph as a unit of currency has undoubtedly been devalued in many disciplines in favour of the journal article, but in the arts and humanities and parts of the social sciences it still holds considerable sway. Writing of scholarship in the area of history, William Savage said: ‘A young scholar, hoping for the kind of job security that begins with tenure and ends with a healthy retirement package, would be foolish to risk writing anything other than a monograph and so says his or her profession.’ [1, p. 484] Being published by a leading university press is an ambition for researchers and the monograph remains central to the work of university presses, as argued by Alison Mudditt when she was Director of the University of California Press:

Monographs are the heart of university press publishing; our fundamental role is to serve as a channel for scholarship that does not have an immediate commercial return. The monograph remains a vital vehicle for scholarly communication in many fields, not to mention the gold standard for promotion and tenure. [2], p. 32]

Yet shrinking library budgets for books, with journals a must-have acquisition, have led to a spiral of lower print runs and higher prices for monographs. Richard Fisher wrote in 2012 about the shift in resources away from AHSS:

The proportion of revenue spent by major research libraries on books, as opposed to serials and data, has declined from around 50 per cent in 1976 to around 15 per cent now. Which implies, inter alia, a massive transfer of net resources from the arts and social sciences to the scientific disciplines; both cause and effect of the significant reduction in the sales and circulation of specific pieces of HSS research that we have witnessed over the past generation. [3], p. 9]

Profitability per copy has been maintained for publishers through higher prices, together with tight control of first costs. Digital printing facilitates low print runs and true print to order, and obviates the need to lock up cash in stock. How long, however, can the spiral of lower sales and higher pricing be maintained? Writing back in 2005, John Thompson observed that ‘The decline in the sales of scholarly monographs has undoubtedly been one of the most significant trends which academic publishers have had to deal with over the last two decades … the unit sales of scholarly monographs have fallen to a quarter or less of what they were in the 1970s’ [4], p. 94] A 2008 study of attitudes amongst researchers to monograph publication reported: ‘One interviewee felt … that the big publishing houses (e.g. CUP) appear to be publishing monographs less and less – preferring more general subject overviews or subject guides’ [5], p. 79]. By 2017, referring to the ‘crisis of the monograph’, Michael Jubb stated that the commentary on the perceived crisis is longstanding and continues to expand [6], p. 46]. He saw ‘an environment where the potential supply of new titles appears greater than the effective demand for them.’ (50).

How should we assess the health of the monograph? The number of titles published seems a good measure and the wellbeing of national publishing industries is often calculated using this metric. Geoffrey Crossick in his 2015 report to the Higher Education Funding Council for England (HEFCE) suggested that the decline in monograph publishing turns out to be something of a myth, pointing to growth in the number of titles published:

Data on new titles were provided for this review by the four largest publishers of monographs in the UK and, although no more than a significant indicator of larger publishing trends, the results for these four major publishers are revealing. They show very significant growth in the numbers of new monograph titles being published by them year-on-year: 2,523 new titles were published by these four publishing houses in 2004, rising to 5,023 new titles in 2013. [7], p. 21]

He concluded that the monograph was still of value, and that ‘The perception that academic books are not being read, or even read in depth, does not appear to be sustained by the evidence.’ (4) Offering data from the US distributor Yankee Book Peddler (now owned by EBSCO), Albert Greco reveals that over the same period the number of scholarly books published in the USA grew from 54,835 new titles in 2004 to 64,709 new titles in 2013. Breaking down the 2013 figure shows that university presses published 11,710 new books (18% of the total) and commercial scholarly publishers produced 52,999 new books (the other 82%) [8], p. 105]. These figures do cover a broader range of books than AHSS monographs, including STM and professional titles, but again show title growth.

There is no doubt that the open access agenda is being extended to long-form research, and for example UK Research and Innovation (UKRI) announced in autumn 2021 that new monographs which acknowledge UKRI funding should be made OA from January 2024, with a preference for immediate open access whilst allowing an embargo period for up to 12 months.

The present research was carried out in 2021 with the distinct aim of examining the position of the AHSS monograph from the point of view of scholarly publishers, covering a number of aspects including the number of titles, revenues, print runs, subject areas and digital developments.

## Methodology

An online survey was sent out by the Oxford International Centre for Publishing in February and March 2021 to leading publishers of monographs with the promise that anonymity would be preserved in the publication of the results. The survey included questions across a range of topics pertinent to the publication of academic monographs. In total 25 university press and commercial publishers responded to the survey, together representing output in 2020 of approximately 32,600 monograph titles in the arts, humanities and social sciences. This is estimated to be approximately 75% of total English language monograph output across the industry. The responding publishers included the larger commercial and university presses as well small and medium-sized ones. Publishers with open access publishing models formed part of the responding group. In order to preserve the anonymity of the respondents and to make the survey as easy as possible to complete, pre-set ranges were given for many of the questions. This meant that for certain questions it was not possible to draw precise figures as to revenue/turnover or publishing output.

The term monograph is commonly used to refer to a long-form academic book on a single research topic, normally written by a single or, on occasions, several authors. Following Crossick [7], included in the survey were edited collections of research essays, critical editions of texts and other works, short-form monographs, academic/trade cross-over titles and other outputs of research other than journal articles. Textbooks and pure trade titles were outside the scope of the research.

There were 25 responses from monograph publishers, separated out as 15 university presses, 9 commercial presses and 1 learned society.

## Monograph Revenues and Output

Five of the nine commercial publishers surveyed, including all commercial publishers with monograph revenues larger than £5 m, publish more than 250 new monograph titles a year. Two, sized between £1 and £5 m, publish 101–250 titles a year; one, with sales of less than £1 m, publishes between 51 and 100 titles a year; and one publishes between 10 and 50 titles.

**Fig. 1:**
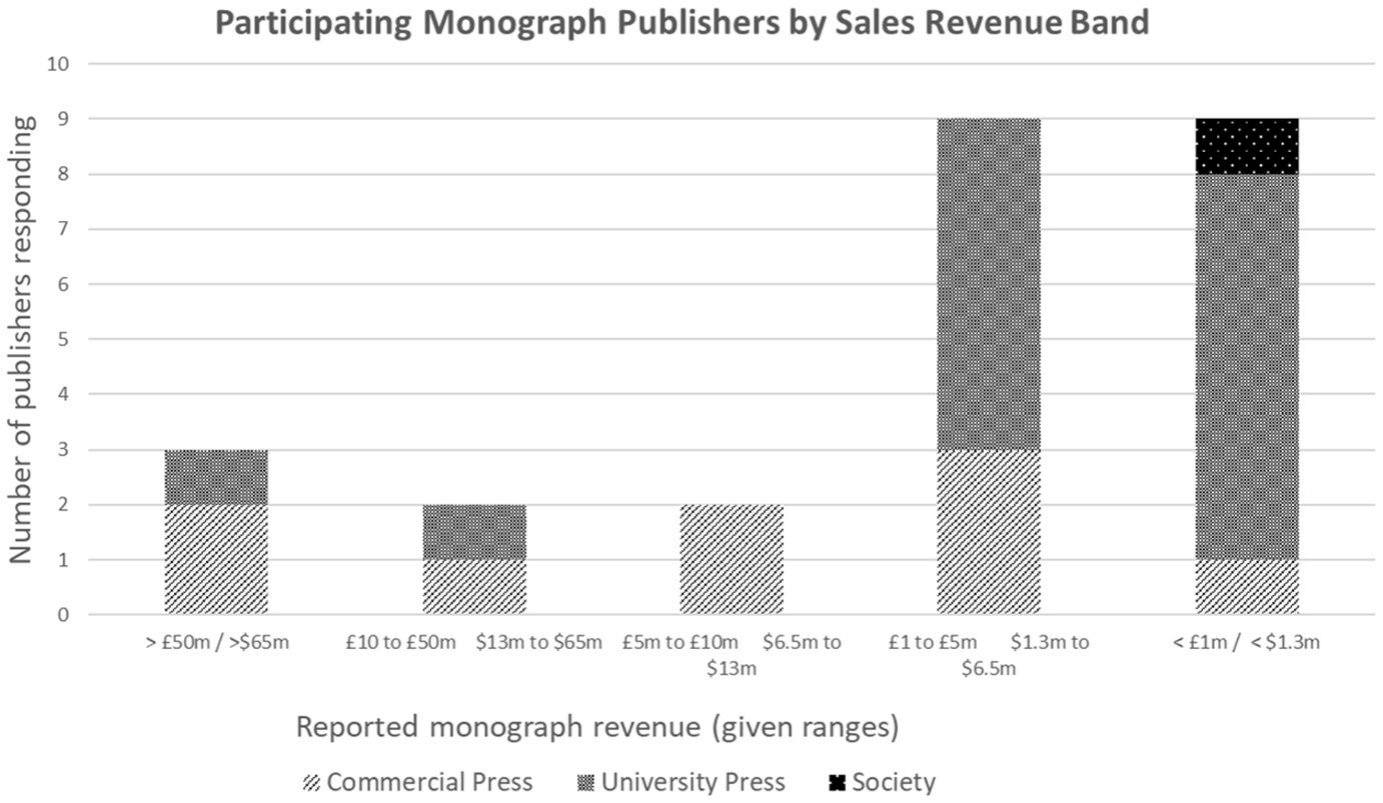
Sales revenues from monographs

**Fig. 2:**
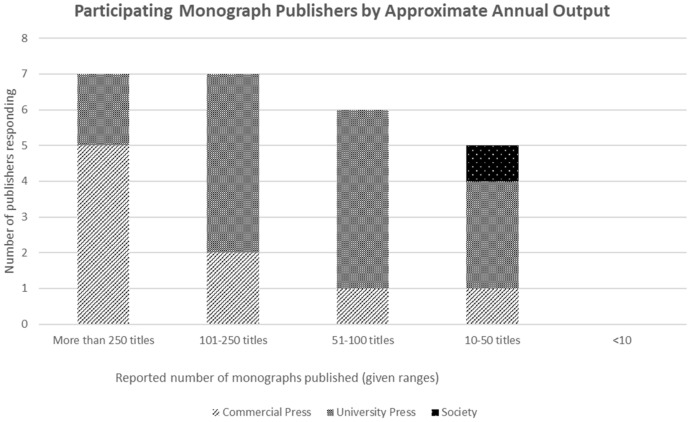
Output of titles

The two university presses reporting monograph revenues above £10 m both publish more than 250 titles. Of the seven university presses sized between £1 and £5 m, four publish 101–250 titles, and three publish 51–100. Of those under £1 m one publishes between 101 and 250, two between 51 and 100 titles and three between 10 and 50 titles (Figs. 1, 2).

Respondents reported on sales revenues in the years from 2017 to 2020. All but two publishers remained in the same sales revenue value band throughout the period surveyed. The two exceptions were smaller university presses that moved between categories.

A medium-sized commercial press commented, ‘Monograph sales grew between 2017 and 2020, largely as a result of growth in the number of monographs we publish annually.’ A second commercial press made a similar observation, ‘We launched a new book programme in 2016 and greatly increased our output from 2018 onwards, hence the increase in sales figures from 2019. In addition, in 2020, due to the coronavirus pandemic, we saw a decline in print sales, as seen across the book industry’. Another large commercial press said, ‘A greater proportion of the monograph revenue now comes from sales of ebooks’. A university press commented, ‘Revenue from print sales has decreased; revenue from ebooks has increased, but not nearly enough to compensate.’

Respondents were asked to indicate, within ranges, their geographical mix of sales. Examining the responses by major geographical region reveals that for most presses the Americas, and we can infer North America predominantly, represent over 25% of total sales, and for most university presses over 35%. The UK, for over half the respondents, represents less than 15% of total sales. One interesting difference between commercial and university presses was the greater importance for the former of sales made in Asia and Australasia. Whereas more than half of the commercial presses replied that Asia and Australasia represent either 25 to 35% or 15 to 25% of total sales, none of the university presses offered a similar response.

**Fig. 3:**
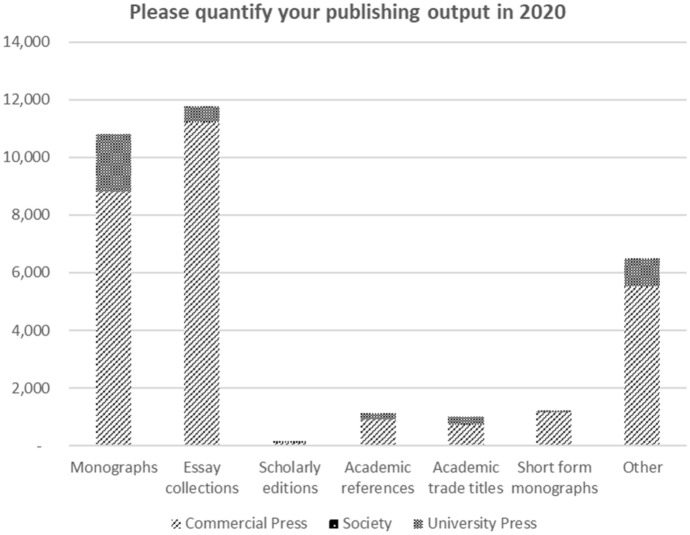
Output by type of publication

Respondents were asked to indicate their publishing output in 2020 and to categorize their title output by type of publication. The total output identified by the 25 respondents was 32,600 titles. Of these the nine commercial publishers released 28,409 titles (87% of the total) and the university presses 4,191 titles (13%). This compares to Greco’s figures above of 82 and 18%. Although data were not collected from all known active publishers, with gaps among both commercial and university presses, it is estimated that the publisher mix represents approximately 75% of monograph output, including the majority of larger presses both commercial and university. Figure 3 presents the analysis of reported output in 2020 showing the most published categories as the traditional monograph and edited collections. We can observe the arrival of the short-form monograph as a significant development although title numbers still remain low.

**Fig. 4:**
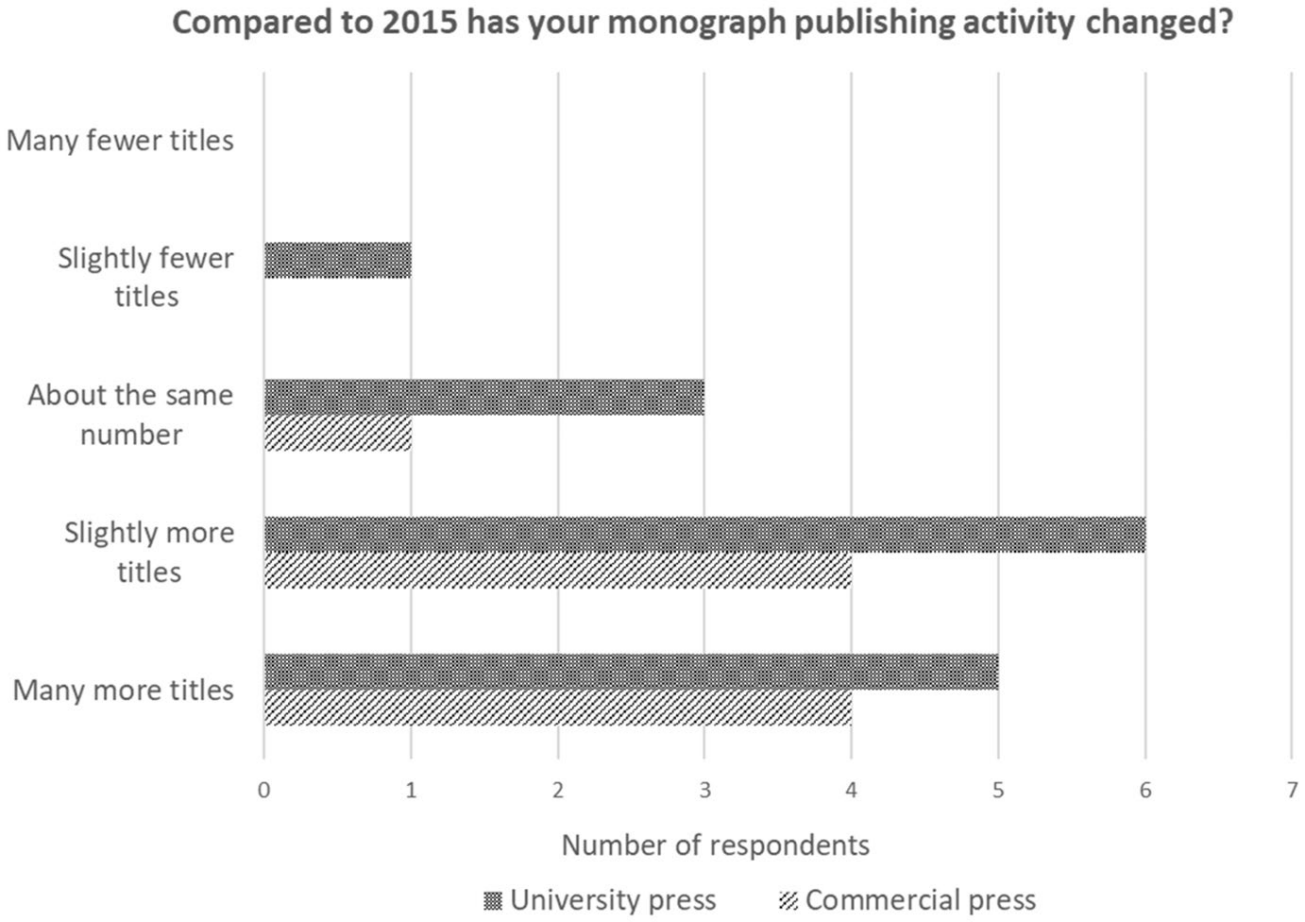
Changes in output of monographs

The survey highlights continued growth in the number of new titles. Respondents were asked, ‘Compared to 2015 has your monograph publishing activity changed?’ Eight out of nine commercial presses indicated that they are publishing more monograph titles than they were in 2015, with the largest commercial presses all indicating ‘many more’. Eleven out of 15 university presses indicated that they are publishing more titles. Whereas the picture across the commercial presses was consistent, regardless of size, the larger university presses indicated ‘About the same’ or ‘fewer’ titles. It was the smaller university presses (smaller than £1 m/$1.3 m) that indicated they are now publishing many more titles than previously (Fig. 4).

Most commercial presses and university presses indicate sales of print copies (whether hardback or paperback) to be below 400 copies per title over the first 3 years, with the majority of commercial presses identifying ‘less than 200’ as the most usual level. Interestingly, the larger commercial presses give ‘less than 200 copies’ as their average volume sale, whereas the larger university presses give ‘200 to 400 copies’.

**Fig. 5:**
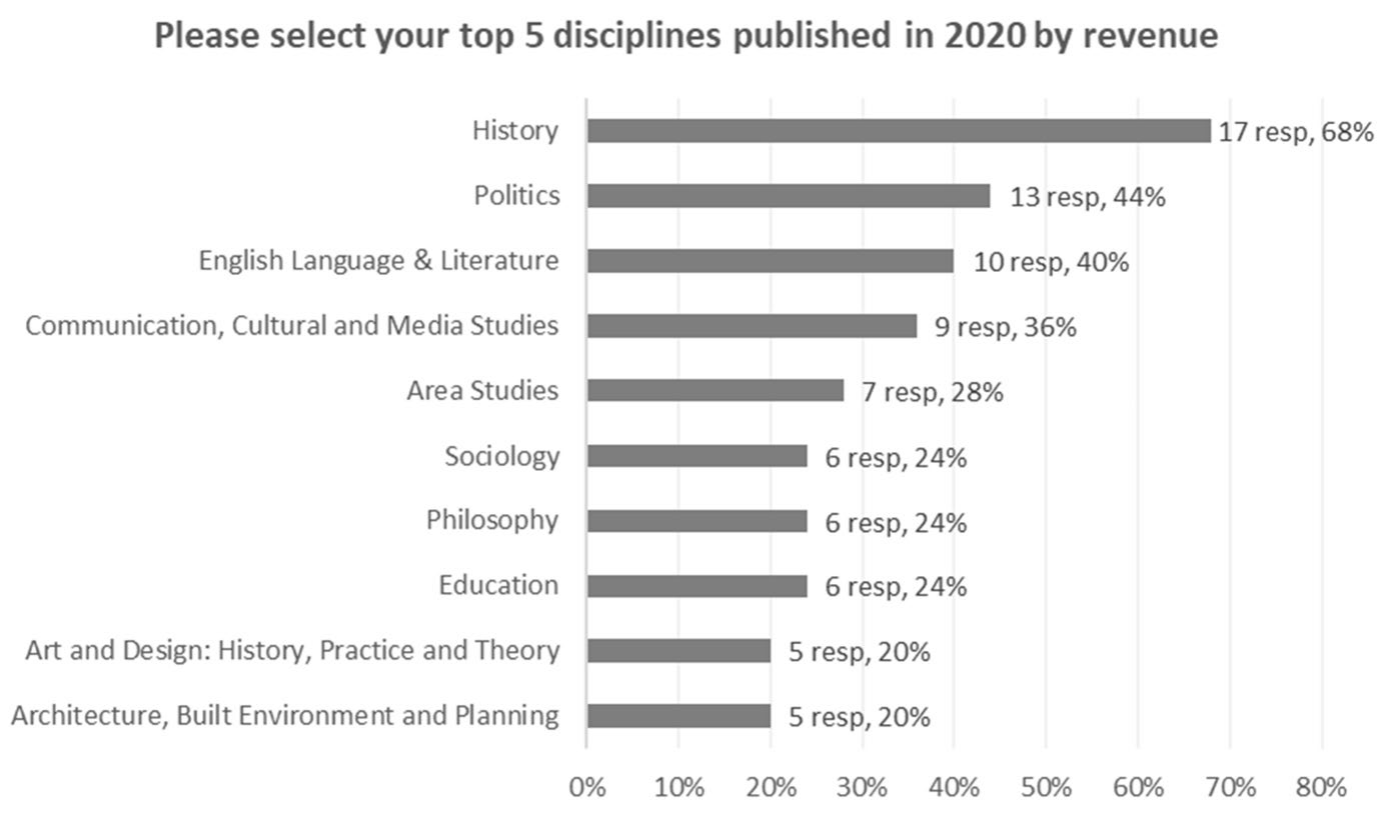
Top disciplines for monograph publication

The survey suggests some trends regarding the subject areas published. Respondents were asked to identify the top five disciplines in their publication of monographs in 2020. Figure 5 presents the results for all respondents. It is not possible to identify specific growth trends by subject but certain disciplines consistently emerged as leading areas among both university and commercial presses. Various ‘applied’ disciplines, including business, economics and education were identified more frequently by the commercial presses; these disciplines were identified much less frequently by the university presses.

A total of 17 of the 25 respondents (68%) placed history among their top five disciplines. This was followed by politics and international studies, identified by 11 respondents (44%), and English language and literature identified by 10 respondents (40%), and communication etc. studies identified by 9 respondents (36%). The frequency with which these disciplines arise suggests that they are perceived by publishers as strong areas for monograph publishing.

We observed some interesting differences between the commercial and the university presses. History, politics/international studies, and English language/literature make consistent appearances as top five disciplines among the 15 university presses (appearing 14, 8 and 8 times respectively). By contrast among the nine commercial presses these three disciplines are less conspicuous (appearing 3, 3 and 3 times respectively). The nine commercial presses identify business, economics, sociology, and education the most frequently (5, 4, 4 and 4 occurrences respectively). These same disciplines are mentioned less often by the 15 university presses (0, 0, 2 and 2 occurrences).

## Pricing

The responses as to monograph pricing reveal some differences between commercial presses and university presses. In general, commercial presses appear to attach higher price points to their titles than do university presses. Although there is a similar pattern in respect of both UK£ and US$ prices it is more noticeable in respect of US$ prices. The indicative hardback volume prices given by the commercial presses yield averages of £87.43 and $120.50; the university press averages are £74.80 and $88.14. For commercial presses the ‘average price per page’ is £0.35 and $0.47; and among the university presses £0.28 and $0.30. The data on volume pricing are summarised in the table below.

**Table:** 

Analysis of monograph pricing		
Commercial presses	Range: £70–£120	Range: $100–$160
	Average: £87.43	Average: $120.50
University presses	Range: £50–£100	Range: $45–$130
	Average: £74.80	Average: $88.14

Recognizing that the pricing of monographs by publishers for institutional library customers is highly dependent on access models and on the composition of the online collections that are available—typically as an aggregated purchase or subscription—the survey asked about the pricing of individual ebooks. Overall 42% of respondents indicated that they apply the same price to digital books as to print. The majority of university presses indicated that they set ebook prices at the same level as the print edition price, using the paperback price (if there is one) as the base. Three of the commercial presses stated that they price at the same level as the print, two indicated ‘less expensive than print’, three indicated that they price at 80% of the print price and one at 90% of the print price.

## Digital Access

In the survey, 21 of the 25 responding publishers indicated that 100% of new monograph titles are now made available in digital form. The remaining four (all university presses) mention that 75% of new monograph titles are available in digital form. Despite the ubiquity of digital monograph distribution, sales in print format continue and are sustained by print-on-demand and short-run digital printing. Most publishers report that more than 75% of their monograph sales are made of books that are printed on demand. Although several comments were made about declining print sales and, as noted previously, digital sales represent a growing proportion of revenues for both commercial presses and university presses, print persists as a format. All but one of the responding publishers indicated that they expect to make available in print form 100% of new monograph titles. One smaller university press indicated that 75% of titles would be available in print. Print on demand (POD), or ultra-short run digital printing, is now used by most monograph publishers. The majority of both commercial and university presses report that more than 75% of print sales are made of copies that are printed on demand.

**Fig. 6:**
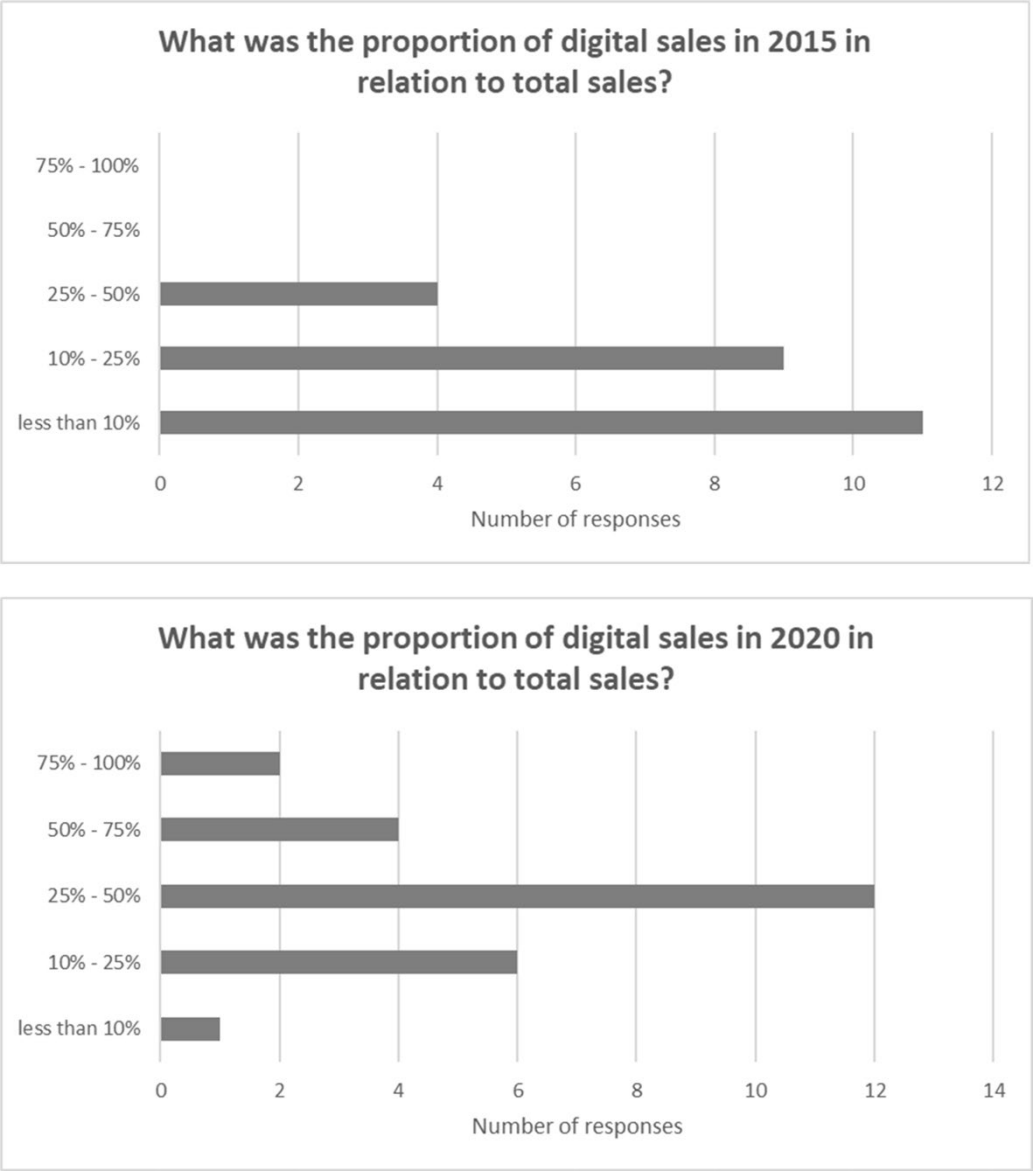
Digital sales of monographs (2015 and 2020)

The shift towards a higher proportion of digital sales among monograph publishers is marked. The survey asked respondents to indicate for 2015 and 2020 the proportion of digital sales in relation to total sales, giving ranges to make responding easier. Figure 6 presents the lower end of the ranges given and may thus under-represent the trend.

The university presses demonstrated a lower proportion of digital sales in both 2015 and 2020 than the commercial presses, but the survey results demonstrate very clearly the overall trend towards more digital sales, with the commercial presses transitioning faster than the university presses. All of the larger commercial presses reported digital sales in 2020 at 50% or more of total monograph sales. Two smaller commercial presses were in the 25 to 50% band. The larger university presses indicated 25 to 50% of revenues from digital in 2020. One small university press reported more than 75% of sales as digital.

We explored whether respondents made available branded collections of their own monographs, asking, ‘Does your organisation include monographs in your own branded digital collection(s)?’ Eight out of the nine commercial presses gave a positive answer to this question, and one reported that it does not make its own branded collection available. Of the university presses, ten replied ‘yes’ and five ‘no’. All respondents distribute their monograph titles through aggregators and library reseller platforms. The most frequently mentioned platforms were EBSCO and Proquest, by both university presses and commercial presses. JSTOR, MUSE, UPSO and Cambridge Core were each mentioned by several. OAPEN was mentioned for the distribution of OA monographs.

## Usage and Open Access

**Fig. 7:**
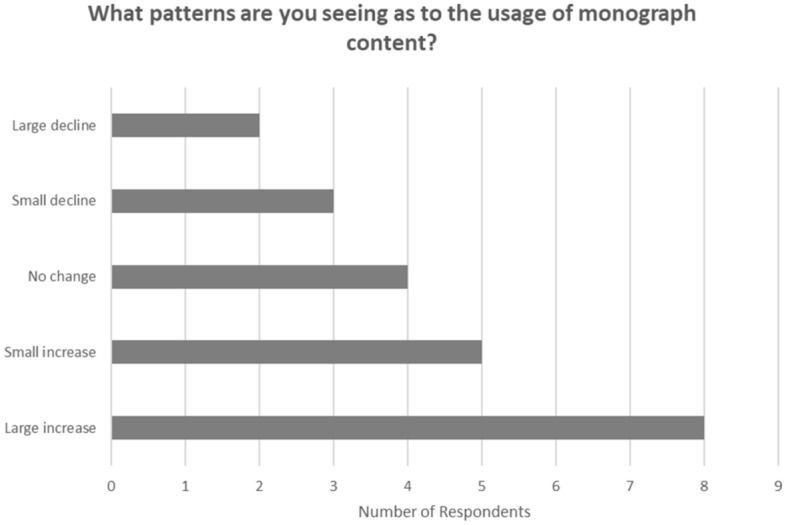
Usage of monographs

Respondents were asked ‘What patterns are you seeing as to the usage of monograph content?’ and were given a range of options for their responses. Over half of those who answered the question indicated an increase in usage as shown in Fig. 7. Increase in usage was more noticeable among the commercial presses. Four of the seven commercial publishers who answered the question indicated a ‘large increase’ in usage and two a ‘small increase’. Of the 14 university presses that answered the question four indicated a ‘large increase’, and two a ‘small increase’. Four indicated ‘no change’ and four either a ‘large decline’ or a ‘small decline’ in usage. We were surprised that nine respondents indicated either ‘no change’ or a decline in usage, expecting that all respondents would have observed an increase in usage.

**Fig. 8:**
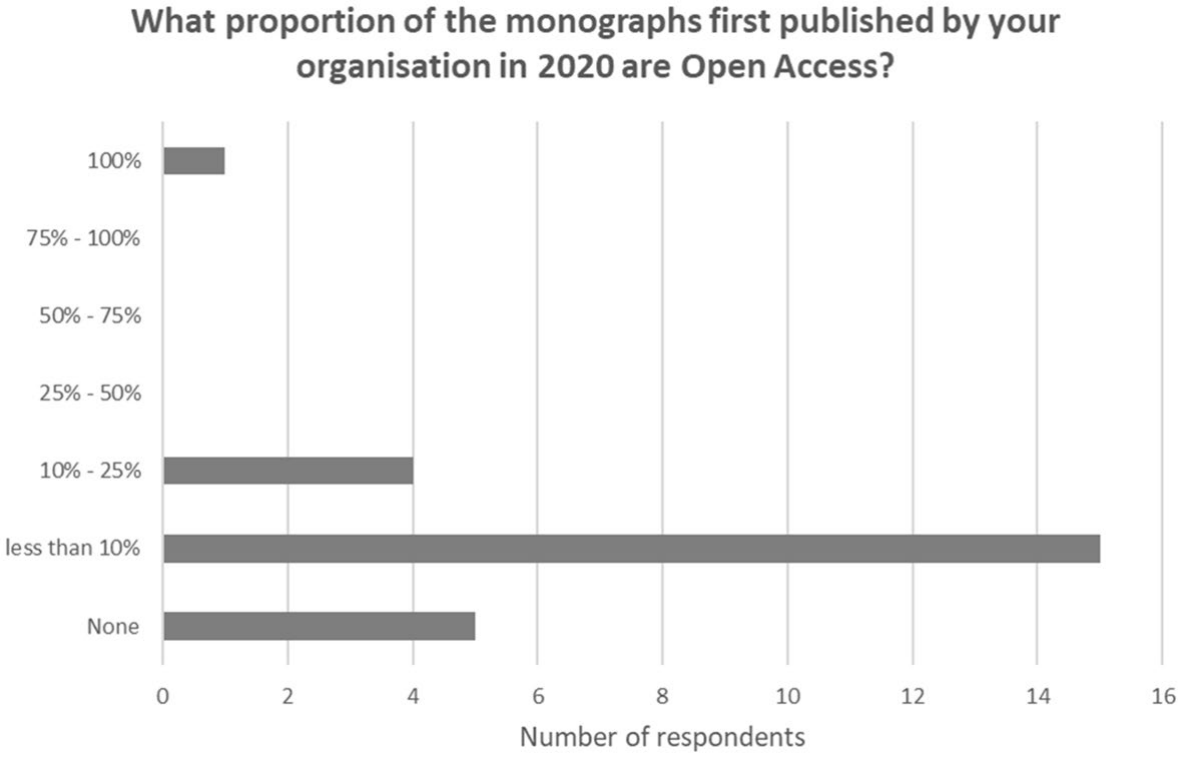
Monographs and open access

Eight out of nine of the commercial presses indicated that fewer than 10% of the monographs they published in 2020 are available open access (OA). One commercial press indicated in the range 10 to 25%. Of the university presses four indicated no OA titles, seven indicated fewer than 10%, and three gave the range ‘between 10 and 25%’. One university press responded 100% OA. Out of the whole sample of 25 presses (see Fig. 8) 20 responded that fewer than 10% of authors have funding for OA publication. Three gave the range of 10 to 25% of authors with funding. One press, with an OA publishing model, indicated that over 50% of authors have funding. The most commonly reported funder of OA publication across the survey is the research funder, followed by the author’s own institution.

## Conclusion

By the measure of the number of titles published, the monograph remains an important vehicle of publication. Most publishers in the research reported an increase in the number of titles published over the period from 2015 to 2020 (this continues the trend identified by Crossick). Much commentary on the decline of the monograph has concentrated on the role of the university press but the research clearly highlights the important role of the commercial publisher. Indeed it appears that commercial publishers are publishing up to seven times as many monograph titles as university presses.

If the number of titles published reveals a reasonably healthy picture, what about sales per title? It can be argued that print sales per title are holding up well within what is long-term decline. Thompson suggests that in the 1970s an academic publisher would print between 2000 and 3000 hardback copies of a monograph. His analysis from 2005 revealed total sales of monographs to be often as low as between 400 and 500 copies per title. [4] The present research shows that the larger commercial presses give their average volume sale over the first 3 years as fewer than 200 copies, whereas the larger university presses give the range of 200 to 400 copies. The top of this latter range coincides with the lower end of Thompson’s figure, although he did comment that publishers often printed more since they could not be certain which books would sell out their print run, and which would not. The arrival of digital printing and printing single copies to order has removed both the need to overprint and, for many publishers, the necessity to hold stock. More than 75% of monograph sales are now made of books that are printed on demand. The lower sales of monographs from commercial publishers are perhaps reflected in their higher pricing: the indicative hardback volume prices given by the commercial presses yield averages of £87.43 and $120.50, the university press averages are £74.80 and $88.14. Thompson [4] wrote that in 2002 it was common for a UK-based academic publisher to price a hardback monograph in the region of £50 to £60—accounting for inflation, a £55 book would by 2020 be priced at £91.50, suggesting publishers have not been raising prices ahead of inflation.

Meanwhile the growth in digital sales should support access and usage. The research shows strong growth in the switch from print sales to digital, and captured the first year of the global pandemic, a time when there was increased demand for digital resources. All of the larger commercial presses reported digital sales in 2020 at 50% or more of total monograph sales. Two smaller commercial presses were in the 25 to 50% band. The larger university presses indicated 25 to 50% of revenues from digital in 2020. All respondents distribute their monograph titles through aggregators and library reseller platforms. Eight out of the nine commercial presses included monographs in their own branded digital collections, alongside ten out of fifteen of the university presses. A majority of respondents reported an increase in the usage of monograph content, although there were a few reports of a decline. Fewer than 10% of monographs new in 2020 were published open access, reflecting low levels of interest from authors and a lack of funding for the gold model in the subject areas populated by monographs.

The top four disciplines across both types of press were history, politics and international studies, English language and literature, and the broad category of communication, cultural and media studies, library and information management. There are differences between the university presses and the commercial publishers. The traditionally strong areas for monographs of history, politics/international studies, and English language/literature feature strongly for the university presses; the areas of business, economics, sociology, and education feature strongly among the commercial presses.

The health of the monograph continues to surprise. The central position of the monograph in academic career paths maintains the supply of new books to publishers. Whilst the research reveals continued decline in print sales of the monograph per title, overall the number of titles shows growth—compensating for the lower sales per title and maintaining the publishing trope that output of titles rises as sales fall. For publishers levels of profitability can be secured through control of first costs, minimizing stock and digital printing to order. Whilst many books are priced beyond the reach of individual readers, digital distribution promotes access and usage levels. Open access models have not yet taken hold as is the case with journal publication, but certainly this is the next development for researchers to study.
